# Adverse event following vaccine surveillance in Kaduna State, Northwestern Nigeria (January 2018 -June 2019): analysis of health facility´s records

**DOI:** 10.11604/pamj.2021.40.268.26961

**Published:** 2021-12-30

**Authors:** Sambo Godwin Ishaku, Gregory Umeh, Bulus Adzu, Anthony Onimisi, Madubu Dauda, Hadiza Aliyu Iyal, Neyu Iliyasu, Danjuma Jenom Sunday, Jeremiah Daikwo, Sannom Mildred Yates, Ibrahim Idris Ibrahim, Lami Hajara Samaila, Basirat Abdullahi, Stephen Kadarko Parom, Kabir Yusuf Maiwashi, Fureratu Zakari, Kase Sarah Nuhu

**Affiliations:** 1 World Health Organization (WHO), Kaduna State Office, Kaduna, Nigeria,; 2 Department of Pharmacology and Toxicology, National Institute for Pharmaceutical Research and Development, Abuja, Nigeria,; 3 World Health Organization (WHO), Abuja, Nigeria,; 4 Kaduna State Primary Healthcare Board, Kaduna, Nigeria,; 5 Ministry of Health and Social Services, Kaduna, Nigeria,; 6 Barau Dikko Teaching Hospital, Kaduna State University, Kaduna, Nigeria,; 7 Faculty of Pharmaceutical Sciences, Kaduna State University, Kaduna, Nigeria,; 8 National Primary Health Care Development Agency, Abuja, Nigeria,; 9 Department of Medical Laboratory Science, Kaduna State University, Kaduna, Nigeria

**Keywords:** Immunization, surveillance, Kaduna State

## Abstract

**Introduction:**

Adverse Events Following Immunization (AEFI) are one of the main reasons for inadequate immunization coverage in Kaduna State, and AEFI underreporting serves as a barrier to achieving goals of global pharmaco-vigilance for vaccine. The purpose of this study is to estimate the completeness of variables in the AEFI line-listing forms, calculate AEFI reporting rates by Local Government Areas & vaccine type and profile the reported cases according to their reactions.

**Methods:**

we conducted a descriptive, cross-sectional, retrospective study of primary surveillance records. We calculated AEFI reporting rates in the State and Local Government areas and AEFI Vaccine reaction rates to the various antigens. We used Binary logistic regression to determine the association between gender and vaccine reactions.

**Results:**

seven thousand eight hundred and twenty-four (7,824) AEFI cases were reported. The completeness of variables on the filled AEFI line-list varied from 21% to 100%. The State had a high AEFI reporting rate of 9.09 per 10,000 administered doses. Fever (<38oC) was the main AEFI reaction. Severe AEFI cases accounted for only 0.89% of the total reported cases. Pentavalent vaccine was the suspect antigen responsible for the highest number of AEFI cases, with a vaccine reaction rate of 44.77 per 10,000 doses. The Zaria Local Government area had the highest AEFI reporting rate, while the Sanga Local Government area had the lowest AEFI reporting rate in the State. The difference between genders in the number of reported AEFI cases was not statistically significant (p>0.05). There were 35% higher odds of occurrence of bleeding among males than among females (aOR: 1.354; P-value: p=.012; 95% CI: 1.070-1.715; Nagelkerke-R^2-^: 0.003). The other reactions were not significantly related to gender.

**Conclusion:**

our study shows a higher occurrence of severe AEFI in subjects undergoing pentavalent vaccine. Thiscaused the highest incidence of AEFI. There was no significant association between gender and AEFI reactions.

## Introduction

Adverse Event Following Immunization (AEFI) Surveillance as defined by World Health Organization (WHO) is an ongoing system of detection, reporting, investigation, data analysis, corrective actions, and evaluation [1,2]. Its importance cannot be underscored in helping to link the causality association between the vaccine administered and the reported event for action [2]. Nigeria have instituted AEFI surveillance into its Expanded Programme on Immunization (EPI), in line with WHO AEFI guidelines, and its AEFI guidelines produced in 2011 were revised in 2018 to meet current challenges [2]. Modules on AEFI have previously been part of the Basic Guide for Routine Immunization training conducted for health workers across the country in 2017 [3]. Kaduna State, in Northwestern Nigeria, has, in this regard, trained 2405 personnel from 1184 public and private health facilities in all its 23 Local Government areas [4].

Several reports and local community surveys have indicated that AEFI is one of the primary reasons for inadequate immunization coverage in Kaduna State [5,6] coupled with the fact that in recent times, Nigeria had introduced new vaccines into the immunization schedules. More are expected in the coming years, thereby making it essential to strengthen AEFI surveillance in the country through timely detection, analysis, and adequate responses to AEFI cases [2]. Nigeria mainly practices a mix of passive and active AEFI surveillance, which involves spontaneous reporting by health workers (HWs) and the general public report any condition that they believe could be associated or related to a vaccine/vaccination [2]. AEFIs underreporting is one of the barriers to achieving the objectives of pharmaco-vigilance of vaccines worldwide [1,7]. Despite some reports in the country of high prevalence (34.9%) of AEFI [8] and 22.1% for pentavalent vaccine [9]. Similarly, a review of Nigerian AEFI national report for 2018 [10] showed Kaduna State as one of the States with sub-optimal reporting of AEFI cases in the country. This calls for the need to evaluate the reporting system in the State. It has been shown that some factors responsible for low reporting are unavailability of electronic reporting (83.6%), unavailability of reporting forms (66.4%), and ignorance (58.2%) [11]. Other reasons are fear by health workers being blamed for AEFI and non-report by caregivers, with caregivers not being aware of reporting processes [12,13]. It has been observed that AEFI reporting forms are rarely filled and when filled, data is incomplete or transmitted through the right channel as required [5]. Though very important, adequate resources and knowledge are not enough guarantees that a surveillance system will function optimally [13,14].

This study was aimed at carrying out a retrospective analysis of AEFI cases in the line-listing forms available at the health facilities across the State. The study's objectives are to i. estimate the completeness of variables in the AEFI line-listing forms ii.to evaluate AEFI reporting rates by local government areas & vaccine reporting rates for the various antigens and iii. profile reported cases according to reaction type and iv. to find out factors that are associated with reaction types. Questions to be asked are i. Does the state report a higher occurrence of serious AEFI than expected? Which of the antigen has the highest reporting rate for AEFI? What factor predicts the type of AEFI events reported? However, to the best of our knowledge, no such study was conducted in Kaduna state. To add to the existing body of knowledge and to fully understand and improve the AEFI reporting system in the State, this study becomes essential.

## Methods

This is a descriptive, cross-sectional retrospective study of primary surveillance records from the 1184 health facilities from the 23 Local Government Areas (LGA) of Kaduna State, Nigeria from January 2018 to June 2019. Hard copies of the health Facility AEFI line-list were retrieved from the 23 Local government areas and entered into Microsoft Excel 2019, where the data were cleaned and screened for duplicates. All available records were considered for this study and it serves as the sample size. Study variables are patient´s identity, gender, address, age, date of last immunization, reaction type, type of AEFI, outcome, suspect vaccine and doses, vaccine and diluent batch number, onset time interval, and reporting date. Data without a patient name, reaction type, and outcome were excluded from the analysis. AEFI reported by all the vaccines in the country immunization schedule for the reporting period were included which are Pentavalent vaccine (Penta), Oral Polio Vaccine (OPV), Hepatitis B Vaccine (HBV), Measles, Pneumococcal Conjugate vaccine (PCV), Tetanus/Diphtheria (Td), Yellow Fever (YF) and Injectable Polio Vaccine (IPV). From data in state AEFI line list, gender was used as the predictor variable while AEFI event types were used as our dependent variables. We used binary logistics regression to determine the association between gender and AEFI event types.

**Data analysis**: data analysis was performed using Microsoft excel 2019 and IBM statistical package for Social Sciences (SPSS) version 25 [15]. The frequency, distribution of the cases, and AEFI reporting rates were analyzed using descriptive statistics. Vaccine reaction rates were calculated using reported AEFI as the numerator, and total administered doses from District Health information system version 2 (DHIS2) as the denominator for the period. AEFI reporting rates for the various LGAs were calculated using the total number of AEFI reported by each Local Government area against the total vaccine doses administered per 10000 doses. Mann-Whitney test was used to compare differences in the number of cases reported by gender with p<0.05 considered significant. The association between gender and AEFI reaction types was determined using Binary-Logistic regression with p<0.05 being as statistically significant.

**Ethics**: ethical approval was obtained from the ethics committee of Kaduna State Ministry of Health to access AEFI records for the period. The approval number for the study is NHREC/17/03/2018 MOH/ADM/744/VOL1/933 dated September 13, 2019.

## Results

All the reports (100%) were from routine immunization and non-recorded from supplemental immunization activities. Seven thousand eight hundred and twenty-four (7824) cases reported in all for the period of study were included in the analysis. Twenty-five records (0.32%) which contains missing mandatory information (AEFI event type, identification of AEFI case , and suspect vaccines) for the line list were discarded and were not included in the analysis as information on the were missing.

**Figure 1:**
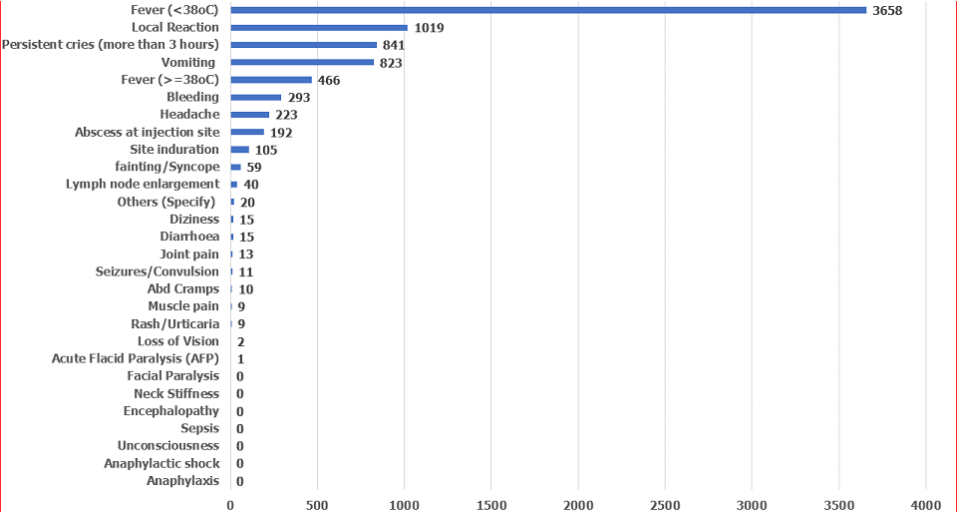
reported AEFI cases by reaction type in Kaduna state (2018-2019)

Completeness of variables on the filled AEFI line-list forms is shown in Table 1, with some variables (Gender, reaction type, outcome, and immunization type) that are 100% completed while the least completed variable is Diluent batch number with only 21% completed. Serious AEFI cases reported are fainting and convulsions (Figure 1) and constitutes only 70 (0.89%) of the reported cases while all other cases were non-serious. No case of hospitalization, disability, nor death was reported as all the cases were reported to have recovered. Table 2shows the frequency of reported AEFI cases by LGAs and AEFI reporting rates per 10000 doses administered in the LGAs, with Zaria LGA having the highest reporting rates in the State while Sanga LGA has the lowest reporting rate per 10,000 doses of vaccines administered. The State has an AEFI reporting rate of 9.09 per 10,000 administered doses.

**Table 1:** completeness of variables in AEFI Line-listing forms in Kaduna State (2018-2019)

Variable	Number (%)
Routine Immunization/SIA_	7824(100%)
Health Facility/Vaccination Post	7711(99%)
Patient's Name	7822 (99%)
Patient's Identity number	3693(47%)
Gender	7824 (100%)
Address	7773(99%)
Age	7423 (95%)
Date of Last immunization (dd/mm/yy)	7359(94%)
Reaction type	7824 (100%)
Type of AEFI (Non-serious or Serious)	7824(100%)
Outcome	7824(100%)
Suspect Vaccine (Name& Dose)	7821 (99%)
Vaccine Batch/lot No	5533(71%)
Diluent Batch No	1669 (21%)
Onset Time interval (hours, days, weeks)	7026(90%)
Date of Reporting (dd/mm/yy)	7201 (92%)

**Table 2:** AEFI reported and reaction rates in Kaduna State (2018-2019)

Local Government Area	Number of reported AEFI (%)	AEFI Reaction rates/10000 doses
Birnin-Gwari	127 (1.62%)	2.78
Chikun	115 (1.47%)	2.47
Giwa	229 (2.93%)	4.63
Igabi	749(9.57%)	11.19
Ikara	270(3.45%)	5.98
Jaba	238 (3.04%)	17.54
Jema´a	65 (0.83%)	2.96
Kachia	105 (1.34%)	4.50
Kaduna-North	796 (10.17%)	22.74
Kaduna-South	523(6.68%)	13.35
Kagarko	330(4.22%)	10.73
Kajuru	483(6.17%)	31.16
Kaura	173 (2.21%)	14.66
Kauru	231 (2.95%)	6.41
Kubau	919 (11.75%)	18.06
Kudan	331 (4.23%)	13.52
Lere	152(1.94%)	2.51
Makarfi	116(1.48%)	4.04
Sabon-Gari	123 (1.57%)	3.10
Sanga	15 (0.19%)	0.75
Soba	418 (5.34%	5.96
Zangon-Kataf	76 (0.97%)	2.72
Zaria	1240 (15.85%)	21.69
State Total	7824(100%)	9.09

Pentavalent vaccine is the suspect antigen responsible for the highest number of AEFI cases (Table 3), accounting for 90.91% of reported cases and a vaccine reaction rates of 44.77 per 10000 doses while the suspect antigen for the lowest number of attributable AEFI cases is Td with only 17 cases being reported for the period representing 0.22% and a vaccine reaction rate of 0.18 per 10000 doses. The distribution of the cases by gender shows that 49.9% (3904) were males, while 50.1% (3920) were female. Using the Mann-Whitney test, there was no statistically significant difference (p=0.9523) between the gender in the number of AEFI cases reported.

**Table 3:** AEFI vaccines reaction rates in Kaduna State (2018-2019)

Suspect vaccine	Total doses administered	Number	Vaccine Reaction rates/10000 doses
BCG	532,399	174 (2.22%)	3.268
HBV	355.964	106 (1.36%)	2.978
MEASLES	513,613	82(1.05%)	1.597
OPV	2,048,289	108 (1.38%)	0.527
PENTA	1,588,133	7110 (90.91%)	44.77
PCV	1,587,779	139 (1.78%)	0.875
TD	942.976	17 (0.22%)	0.18
YF	504,414	37 (0.47%)	0.734
IPV	533,181	47(0.61%)	0.9
Total	8,606,748	7821 (100%)	9.087

Twenty-one (21) of the 28 reaction types in the line-list was reported (Figure 1) with fever (<38^o^C) as the main reaction types reported while no single case of anaphylaxis, anaphylactic shock, facial paralysis, neck stiffness, encephalopathy, sepsis, or unconsciousness were reported. Of the twenty-Eight AEFI reaction types studied, gender was only significantly associated with bleeding (Table 4). There were 35% higher odds of occurrence of bleeding among males than among the female gender (AOR: 1.354; P-value: p=.012; 95% CI: 1.070-1.715; Nagelkerke-R^2^: 0.003).

**Table 4:** association between gender and AEFI reaction type in Kaduna State (2018-2019)

Variable	Male				Female
	AOR	P-value	95% CI	Nagelkerke-R2	
Fever (<38°C)	0.930	P = 0.110	0.851-1.017	0.000	Ref
Local Reaction	0.950	P = 0.443	0.833-1.083	0.000	Ref
Persistent cries (more than 3 hours)	1.037	P = 0.622	0.898-1.196	0.000	Ref
Vomiting	1.150	P = 0.058	0.995-1.330	0.001	Ref
Fever (≥38°C)	0.893	P = 0.236	0.740-1.077	0.000	Ref
Bleeding	1.354	P = 0.012**	1.070-1.715	0.003	Ref
Headache	1.280	P =0 .071	0.979-1.674	0.002	Ref
Abscess at injection site	0.934	P = 0.643	0.702-1.245	0.000	Ref
Site induration	0.959	P = 0.830	0.654-1.407	0.000	Ref
Fainting/Syncope	0.784	P = 0.354	0.468-1.313	0.001	Ref
Lymph node enlargement	1.102	P = 0.760	0.591-2.053	0.000	Ref
Others	0.996	P = 0.994	0.414-2.397	0.000	Ref
Dizziness	1.496	P = 0.445	0.532-4.206	0.003	Ref
Diarrhoea	3.992	P = 0.080	0.847-18.81	0.025	Ref
Joint pain	0.854	P = 0.777	0.287-2.543	0.000	Ref
Seizures/Convulsion	1.196	P = 0.768	0.365-3.922	0.001	Ref
Rash/Urticaria	0.498	P = 0.324	0.124-1.992	0.007	Ref
Muscle pain	0.797	P = 0.735	0.214-2.970	0.001	Ref
Loss of vision	0.000	P = 0.983	0000-0000	0.075	Ref
AFP	0.000	P = 0.984	0000-0000	0.070	Ref

** Significant (p < 0.05)

AFP: Acute Flaccid paralysis

## Discussion

A retrospective review of health facilities records and has been shown to be appropriate for the study of health events [1,16]. However, this has enabled us to estimate approximate AEFI reporting and vaccines reaction rates as well as and to assess the performance of passive AEFI surveillance in Kaduna State. Hence, it is considered fit for this study. Therefore, for this study, the number of administered doses was used against surviving infants as our denominator and was documented as the most reliable when available compared to population-based, which is subject to larger variabilities when accurate population figures are not available [1].

There were substantial data quality issues (Table 1) as not all the fields on the line-list were filled with the required information. While six of the fields in the line-list were completely filled, six other fields were between 90% & 99% filled, while vaccine batch number and diluent batch number were observed to be poorly filled. It can thus be suggested that health workers might not have seen the necessity of completing this two information and might indicate inadequate knowledge of WHO mandatory core variables that were to be filled as part of AEFI documentation [1]. Previous training on AEFI in the State has not been detailed enough and might have been responsible for this knowledge gap [4]. This is important as it could affect the outcome of the analysis made on suspect vaccines and may likewise make identification of the level of clustering of non-serious AEFI difficult. Thus, the data has not met the standard set by WHO for 100 % mandatory core variables to be reported [1].

Based on Nigeria AEFI guidelines, 28 reaction types were included in the surveillance system, and for this study, this could be seen in Figure 1, a substantial number (which represents 99%) of reaction types were non-serious while less than one percent of the cases reported were serious AEFI. WHO AEFI guidelines described the occurrence of serious AEFI as very rare, when it occurs with a frequency of less than 0.01% [1]. Our study shows the occurrence of serious AEFI was rare based as it lies between 0.01 and 0.1% of the total reported cases based on WHO classification [1]. It has been shown that fear of been blamed was among the factors that influences the reporting of AEFI by health workers [12,13]. This might also be true for our setting, but this needs further investigation. We observed high AEFI reaction rates for the State (Table 2) compared with a study in Zimbabwe [17] where AEFI reaction rates were found to be 0.058 per 10,000 doses. Our study was close enough to the report of Khazei *et al*. [18] in a study in Iran reported where the vaccine rate observed was 11.8 per 10,000 doses. Such high reported cases could indicate issues with quality of service delivery by health care providers in the State. This provides further research opportunities to explore and assess vaccination skills and quality of service delivery.

The pentavalent vaccine was observed to be the main suspect in causing AEFI (89%) of all the reported cases (Table 3), while Td was the vaccine with the least reaction rates. Our present findings were different from a study in Brazil of AEFI due to immunization errors where BCG was found to cause the highest incidence of AEFI in under 1-year-old [19]. However, our findings were consistent with a similar study conducted in Brazil, where the DTP-containing vaccine was found to cause the highest incidence of AEFI [20]. Based on WHO classification of occurrence of reported AEFI [1], the frequencies of occurrence of AEFI by BCG, HBV, and measles antigens are rare (between 0.01% and 0.1%) except for Penta where the frequency of occurrence was uncommon (between 0.01 and 1%) while the occurrence of IPV, yellow fever, Td, PCV, and OPV are infrequent with the frequency of occurrence. Furthermore, our findings also show that gender was not a factor in the number of AEFI cases reported nor was it significantly associated with the reaction types except for bleeding where there was a significantly higher odd of occurrence among males than female though with a low R-squared value. This shows that even with this outcome, less of the observed variation can be fully explained. The twenty-seven other reaction types were not significantly associated with gender. One strength of this study is the availability of a large pool of data to make for meaningful analysis.

**Limitations**: the study has several limitations that the data presents the possibility of ambiguity in the suspect vaccine for vaccine administered together with the causative antigens for AEFI, bearing in mind that more than one antigen is administered during a single visit. Data quality issues like Incompleteness of the variables ‘batch number of vaccines and diluent´, ‘non-presentation of vaccines dose number´ makes it difficult, to identify possible clustering of cases. The study did not classify the cases into various age groups. The study considered only primary data (line list form) as reporting and investigation forms were not available for further analysis.

## Conclusion

Our study shows a higher occurrence of serious AEFI than expected with pentavalent vaccine found to cause the highest incidence of AEFI. There is no significant association between gender and AEFI reaction type reported. The study has shown that the use of primary AEFI data could be useful in estimating vaccine reaction rates/ reporting rates even with a passive AEFI surveillance system in place. In the light of this study, we recommend that a prospective study for vaccines reaction rates, especially for newly introduced vaccine need to be carried out to get an actual incidence of AEFI for the State. Similarly, steps should be taken to stimulate active AEFI surveillance in the State through training of health workers on active AEFI surveillance and analysis as well as provision of AEFI data tools and guidelines to all health workers in the State, and ensure regular and complete reporting of AEFI cases on the DHIS 2 platform.

### What is known about this topic

- *Prior to this study, there were scanty reports of AEFI cases for the State at the national data base;*
- *AEFI was one of the primary reasons for low, inadequate immunization coverage in Kaduna State*.

### What this study adds

- *The State has a high AEFI reporting rate per total administered doses;*
- *The study showed that pentavalent vaccine was found to cause the highest incidence of AEFI events;*
- *Gender was not a predicting factor to the AEFI reaction type reported*.
